# Hemoglobin concentration is associated with self-reported disability and reduced physical performance in a community dwelling population of nonagenarians: the Mugello Study

**DOI:** 10.1007/s11739-017-1762-1

**Published:** 2017-10-25

**Authors:** Francesca Cecchi, Silvia Pancani, Federica Vannetti, Roberta Boni, Chiara Castagnoli, Anita Paperini, Guido Pasquini, Francesco Sofi, Raffaele Molino-Lova, Claudio Macchi, Mugello Study Working Group, Annamaria Gori, Nona Turcan, Debora Valecchi, Roberta Frandi, Lorenzo Razzolini, Valentina Fabbri, Guglielmo Bonaccorsi, Chiara Lorini

**Affiliations:** 1 Don Carlo Gnocchi Foundation, IRCSS, Via di Scandicci, 269, 50143 Florence, Italy; 2 Department of Experimental and Clinical Medicine, University of Florence, Florence, Italy; 3 University of Florence, NEUROFARBA, Florence, Italy; 4 Department of Health Science, University of Florence, Florence, Italy

**Keywords:** Hemoglobin, Anemia, Physical performance, Handgrip, Nonagenarians, Oldest old

## Abstract

People aged 90 and older represent a fast-growing population segment who deserve specific attention and research. Aging is associated with a progressive decrease in hemoglobin concentration, which predicts adverse outcome, such as mortality, morbidity, frailty and disability. Whether this association is independent from increased prevalence of comorbidity, causing both anemia and reduced physical function is yet under debate. The aim of this study is to explore the relationship between hemoglobin concentration and self-reported disability and reduced physical performance in a community dwelling population of nonagenarians. Data presented were collected in the framework of the Mugello Study, a clinical epidemiologic survey of nonagenarians living in the Mugello area (Tuscany, Italy). 251 persons (177 women, age 93.2 ± 3.3 years; 74 men, age 92.2 ± 2.5 years) underwent a blood draw. Along with hemoglobin concentration, self-reported disability (basic and instrumental activities of daily living), physical performance (Short Physical Performance Battery), self-reported physical activity and muscular strength (handgrip measurement) were assessed. Covariates, inherent sociodemographic and health indicators and comorbidities were also included in the analysis. This study confirms that anemia is very common in the oldest old, with a significantly higher prevalence in males (50% in men vs 24% in women). Multiple linear regression analysis, including all the comorbid conditions as confounding factors, shows that hemoglobin concentration is independently associated with handgrip strength, self-reported physical activity and disability in dressing, and taking a shower/bath. In conclusion, results from this study confirm that in the oldest old, low hemoglobin concentration is strongly associated with self-reported disability and decline of physical performance independent of comorbidity.

## Introduction

As the world population is constantly aging, the oldest old, often defined as those aged 85 + or 90 + , present the largest increase in numbers [1]. The saying “the nineties are the new eighties,” [2] well expresses the socio-epidemiological and clinical relevance of this trend. Addressing modifiable manifestations of aging that are relevant to function and quality of life is one of the main goals of geriatric medicine, and persons aged 90, besides being simply older, are a somewhat selected population who deserve specific attention and research [3, 4]. Thus, the identification and treatment of those medical conditions that impact on function and quality of life in the oldest old becomes of critical importance for healthcare provision.

Aging is often associated with a progressive decrease in hemoglobin concentration and erythrocyte indices, due to several factors including decreased erythropoietin secretion, reduced hematopoietic reserve, reduced iron and vitamin intake, and morbid conditions such as chronic inflammation [3]. Although reports of incidence and prevalence of anemia in the elder population are rather discrepant, a steeper decrease of hemoglobin concentration values in the oldest old (age range 85 +) has been observed [5].

Anemia, or even mildly low hemoglobin concentration above the WHO threshold for anemia (i.e., 120 g/l for women and 130 g/l for men) consistently predict worse outcome, including mortality, hospitalization, and falls in the elderly population [5–9]. Recent studies also suggest an independent association in old age between anemia and frailty, and disability [10–13]. However, most of these studies include persons above 60 or 65, while less is known about the oldest old. A paramount issue is whether adverse physical and functional outcomes in this specific share of the population are independently associated with reduced hemoglobin concentration, or rather, as suggested by a recent large Dutch study on the 85 + population [14], they are mainly explained by highly prevalent comorbidity, causing both anemia and reduced physical function.

The Mugello Study [15] is a clinical epidemiologic survey of nonagenarians living in the Mugello area, north-eastward of Florence, Tuscany, Italy. This area is characterized by a higher percentage of nonagenarians with respect to the entire population, 1.76%, compared to the Italian reference value, 1.14% (ISTAT—Data 2015).

The purpose of the present study is to investigate if and how hemoglobin concentration is related to a reduced physical performance and disability in a community dwelling population of nonagenarians.

## Methods

### Participants

The analysis presented in this paper is based upon data from the Mugello Study, a survey on nonagenarians living in the Mugello area, north-eastward of Florence, in Tuscany, Italy, whose detailed description is reported elsewhere [15]. Briefly, all community dwelling persons aged 90 + living in the Mugello area who accepted to be enrolled, underwent a structured interview and medical visit, along with a series of instrumental assessments at their home. Blood samples were also collected to perform routine laboratory tests, and to create a biological bank with serum and plasma aliquots. The Mugello Study protocol, which complied with the principles of the Declaration of Helsinki on clinical research involving humans, was approved by the Ethics Committee of the Don Gnocchi Foundation, and the participants, or their legal representative, signed the informed consent form to be included in the study, and to undergo blood withdrawal.

Among the Mugello Study participants, 98 (74 F mean age 93.5 ± 3.8—24 M mean age 93.1 ± 3.7) subjects refused to provide a blood sample, and were thus excluded from this study, while 251 subjects (177 F mean age 93.2 ± 3.3—74 M mean age 92.2 ± 2.5) had completed data to be included in this analysis.

### Main variables

Hemoglobin concentration, g/dl, was analyzed using the hematology analyzer Sysmex XT-1800 (Sysmex, Inc., Mundelein, IL). Hemoglobin concentration values above 16.5 g/dl (six cases) were excluded from further analyses since high blood viscosity was considered a sign of underlying serious clinical condition [3].

Disability was assessed according to the Katz questionnaire [16] concerning activities of daily life, six basic (eating, showering, dressing, transferring from bed to chair, using the toilet, and continence) and eight instrumental activities (shopping, doing light housework, preparing meals, managing money, using the telephone, taking medications, using transportation, doing laundry) [17]. The score for each activity ranged from 0 to 4; 0 no functional limitation and 4 total dependence. A score over or equal to 2 was assumed as an index of disability. Together with the dichotomic variable created for each activity, the sum (nADL) of reported basic disabilities (BADL) and instrumental disabilities (IADL) was retained for further analysis.

Physical performance was measured using the Short Physical Performance Battery (SPPB) [18], which assesses the walking speed, standing balance and ability to raise from a chair. The total score (SPS), developed by summing the partial scores of each task, ranges from 0, in case a participant is not able to complete one test, to 12, best performance.

Level of physical activity (SPA) was investigated and scored by administering a questionnaire to each nonagenarian. This questionnaire, which has been described elsewhere was modeled on the Harvard Alumni Questionnaire, and adapted for Italian people [15].

Muscle strength was evaluated by means of handgrip force measurement, expressed in kg, using a hydraulic dynamometer (RO + TEN, Verano Brianza, Italy) [19]. Participants repeated the test twice for each hand, and the best value for each side was recorded. In this analysis, the mean value of the best right and left results was used.

In the analysis, covariates, inherent socio-demographics (age, sex) and health indicators were included. Body mass index was calculated as the ratio between weight (kg) and the square of height (m). Comorbidities, such as myocardial infarction, diabetes mellitus, cerebrovascular disease, peripheral artery disease, congestive heart failure, chronic obstructive pulmonary disease, chronic kidney disease, cancer, and gastric ulcer, also considered as dichotomous variables, were determined using an adjudication process that includes self-reported history, medical examination data, and medical records information [15].

Serum levels of high-sensitivity C-reactive protein (hs-CRP) were measured according to WHO First International Reference Standard, with a sensitivity of 0.08 μg/ml using an enzyme-linked immunosorbent assay (ELISA), and a colorimetric competitive immunoassay that used purified protein and polyclonal anti-CRP antibodies; serum creatinine level was assessed using a standard creatinine Jaffe method (Roche Diagnostics, GmbH, Mannheim, Germany), which had an inter-assay coefficient of less than 2.5% [15].

### Statistics

Data are reported as percentage or as mean ± standard deviation. The analysis was performed using SPSS 23.0 software (IBM SPSS, Chicago, IL, USA).

Because of a high skewness some variables were transformed to approximate a normal distribution. A logarithmic transformation was applied to the variables C-reactive protein and creatinine, while a square root transformation was applied to the variable handgrip.

The relationship between hemoglobin concentration and variables that describe disability and physical performance was investigated using a multiple linear or a logistic regression as appropriate, corrected by age and gender.

Similarly, multiple linear/logistic regression analyses were conducted to assess whether the effect of HC level on physical and performance parameters was still significant after adjustment for those comorbid conditions that were shown to be associated with hemoglobin concentration with a *p* value at least < 0.05.

## Results

Participants’ functional and physical characteristics are summarized in Table 1.

**Table 1:** Participants’ characteristics expressed as mean ± SD or as a percentage as appropriate for continuous or ordinal variables

	Mean ± SD (%)
Age (years)	92.9 ± 3.1
Gender (%woman)	71
Hemoglobin (g/dl)	12.8 ± 1.5
Handgrip (kg)	14.6 ± 7.0
BMI (kg/m^2^)	25.5 ± 4.7
SPS (score)	2.9 ± 3.8
nADL (score)	5.5 ± 4.5
Score physical activity (score)	0.7 ± 0.9
BADL (%Y)	
Eating	7
Dressing	40
Taking a shower	63
Using the toilet	31
Moving from chair to bed and vice versa	29
Incontinence	62
IADL (% Y)	
Using the telephone	29
Shopping	57
Cooking	39
Doing light housework	41
Doing heavy housework	68
Doing laundry	50
Using public transport	48
Managing medication	49
Keeping track of finances	43
Washing	18
Walking upstairs	42
Move from one room to another	28
Walking on a flat floor	29

Although hemoglobin concentration data were substantially identical in men (*n* = 74; mean HC 12.85 ± 1.64) and women (*n* = 177; mean HC 12.84 ± 1.43), the prevalence of anemia according to the WHO different cut offs for men and women was much higher in men (50% of men vs 24.3% in women), with an overall prevalence of 31.9%. After adjusting for age and gender, associations between hemoglobin concentration and disability and physical performance are shown in Table 2. Handgrip strength, SPS score, nADL score and SPA are significantly associated with HC level, while there is no association BMI and hemoglobin concentration. When BADL and IADL activities are individually investigated, a significant association between hemoglobin concentration and dressing, taking a shower, doing laundry and using public transport activities is observed (Table 2). Comorbidities significantly associated with hemoglobin concentration are severe kidney disease and congestive heart failure (Table 3). Similarly, a significant association emerges between hemoglobin concentration and creatinine levels, while no association is observed with the C-reactive protein (Table 3).

**Table 2:** Multiple linear and logistic regression model adjusted for age and sex showing the association between hemoglobin concentration and variables that describe physical performance and disability

Hemoglobin concentration (g/dl) adjusted for age, sex	*β* ± SE (*β*)	*R* ^2^	OR	*p*
Handgrip_tr (kg)	0.079 (0.031)	0.402		0.010
BMI (kg/m^2^)	− 0.227 (0.199)	0.013		0.253
Sps (score)	0.361 (0.158)	0.097		0.023
nADL (score)	− 0.384 (0.193)	0.176		0.048
SPA (score)	0.324 (0.155)	0.108		0.037
BADL				
Eating			0.854	0.350
Dressing			0.790	0.014
Taking a shower			0.791	0.016
Using the toilet			0.904	0.318
Moving from chair to bed and vice versa			0.861	0.136
Incontinence			1.063	0.513
IADL				
Using the telephone			1.007	0.944
Shopping			0.916	0.330
Cooking			0.866	0.129
Doing light housework			0.884	0.178
Doing heavy housework			0.925	0.419
Doing laundry			0.830	0.042
Using public transport			0.836	0.047
Managing medication			0.860	0.104
Keeping track of finances			0.868	0.135
Washing			0.839	0.125
Walking upstairs			0.864	0.120
Move from one room to another			0.914	0.369
Walking on a flat floor			0.880	0.195

**Table 3:** Multiple linear and logistic regression model adjusted for age and sex showing the association of hemoglobin concentration with variables that describe the presence of comorbidities and creatinine and C-reactive protein levels

Hemoglobin concentration (g/dl) adjusted for age, sex	*β* ± SE (*β*)	*R* ^2^	OR	*p*
Cardiovascular disease			0.934	0.423
Cerebrovascular disease			1.172	0.127
Myocardial infarction			0.983	0.890
Chronic pulmonary disease			0.845	0.136
Peripheral vascular disease			0.875	0.216
Severe kidney disease			0.572	0.005
Diabetes			0.802	0.059
Cancer			0.943	0.611
Ulcer			0.848	0.137
Congestive heart failure			0.818	0.045
Creatinine _tr	− 0.092 (0.014)	0.200		< 0.001
C-reactive protein _tr	− 0.090 (0.048)	0.020		0.064

When the multiple linear regression analysis was performed including all the comorbid conditions associated with hemoglobin concentration (*p* < 0.05 in Table 3) and the creatinine levels as confounding factors, hemoglobin concentration alone explains the 43% of the variance in the handgrip strength measured and shows a significant association with SPS, nADL and SPA scores (Table 4). The results of regression analysis for those BADL and IADL that showed a *p* value < 0.05 in Table 2, adjusted for age, gender, comorbidities and creatinine indicate that as hemoglobin concentration increases, the odds of developing a disability in dressing and taking a shower activity decrease (Table 4).

**Table 4:** Multiple linear and logistic regression model adjusted for age, sex, comorbidities and creatinine level showing the association between hemoglobin concentration and variables that describe physical performance and disability

Hemoglobin concentration (g/dl) adjusted for age, sex, comorbidities, creatinine	*β* ± SE (*β*)	R^2^	OR	P
Handgrip_tr (kg)	0.118 (0.033)	0.426		< 0.001
Sps (score)	0.348 (0.169)	0.111		0.040
nADL (score)	− 0.484 (0.210)	0.182		0.022
Score physical activity (score)	0.094 (0.037)	0.137		0.013
BADL				
Dressing			0.760	0.009
Taking a shower			0.769	0.012
IADL				
Doing laundry			0.838	0.076
Using public transport			0.847	0.092

## Discussion

This study confirms that anemia is very common in the oldest old share of a community dwelling sample of Italian nonagenarians, with a significantly higher prevalence in men. Although hemoglobin concentration data is substantially identical in men (*n* = 74; mean hemoglobin concentration 12.85 + 1.64) and women (*n* = 177; mean hemoglobin concentration 12.84 + 1.43), the prevalence of anemia according to the WHO different cut offs for men and women are much higher in men (50% of men vs 24.3% in women), with an overall 31.9% who fulfill the WHO criteria for anemia. These results are consistent with other reports on persons aged 85 and older (Leyden), and show a substantial increase of the anemic population compared to studies conducted on younger old patient samples [10, 20].

The appropriateness of the criteria for anemia set by the World Health Organization (WHO), i.e., hemoglobin level < 12.00 g/l for women and < 13.00 g/l for men has been repeatedly questioned for older populations, as even hemoglobin just above the WHO anemia criteria is consistently associated with adverse outcome in older persons [5, 11, 21]. This is why in this study we chose to analyze the relationship of hemoglobin concentration with reduced physical performance, identified by two performance measures such as handgrip strength, which has been shown to be a proxy of muscle strength in the elderly [22], an early marker of poor mobility [19] and mortality in the oldest old [23] and with the Summary of Performance Score, whose lower scores are a powerful predictor of nursing home admission, hospitalization, and disability [24] while SPS higher scores are highly sensitive in discriminating those older persons who are not likely to experience adverse outcomes [25]. Indeed, SPPB might be used to assess frailty, by identifying subjects with a SPPB score of 9 and below. According to this cut-off value, most of our participants (77%) are frail. We also verify the association of hemoglobin concentration with self-reported disability in basic and instrumental ADL and with self-reported physical activity, which in turn predicts subsequent disability even in the oldest old [26, 27]. Our results confirm a significant association between hemoglobin concentration and either Handgrip strength, SPS score, nADL score, as already reported elsewhere both for the younger and for the older old [10–12, 14]. The activities significantly associated with hemoglobin concentration in the regression analysis are dressing and taking a shower, which from a ADL hierarchy perspective correspond with a moderate stage disability [27].

So, as we confirm that even in the oldest old, low hemoglobin concentration is associated with self-reported disability and reduced physical performance, we then attempted to clarify the issue of whether this association is explained by underlying comorbidities that are highly prevalent in the oldest old. Since anemia and low hemoglobin concentration in old age are reported to be significantly associated and in large part etiologically related to chronic conditions such as cancer, renal failure, gastric ulcer and infection [5, 10], we first verified the association of hemoglobin concentration and these conditions in our study population. Indeed, we find that severe kidney disease and creatinine levels, as well as congestive heart failure are significantly associated with hemoglobin concentration, while we find no association with cancer, gastric ulcer and with the C-reactive protein, selected as a marker of inflammation/infection. This is a rather unexpected finding that may be at least partially explained by the high prevalence of these conditions in our study sample, 50% in men and 24% in women, compared to 11.1 and 11.5% observed in men and women, respectively, by Penninx et al. [10].

However, when we performed a multiple linear regression analysis including all the comorbid conditions associated with hemoglobin concentration as confounding factors, hemoglobin concentration level alone remains a powerful predictor of handgrip strength, and maintains a significant association with SPS, nADL and SPA scores. Thus, in contrast with what is reported in a large Dutch study on the 85-plus population, in our sample, low HC levels are associated with reduced self-reported physical activity, and are independently associated with reduced strength, physical performance, physical activity and disability.

The independent effect of hemoglobin concentration on physical functioning, as already reported in other relevant studies conducted on elderly population, may have several explanations. The impact on muscle strength may be related to a reduced muscle oxygenation [25]. Our results do not seem to confirm the hypothesis that the underlying cause both for anemia and for functional decline in the very elderly may be connected with chronic inflammation [10, 28], as we did not find a significant association between low HC levels and CRP. On the other hand, the pattern of disability associated with low hemoglobin concentration is different from that found in other age-associated conditions, such as musculoskeletal pain [29, 30], although reduced SPS scores are similarly found [31, 32]. Thus, the underlying causes leading to disability are probably related to specific mechanisms, such as for instance fatigue [10, 28], which may be increased by disuse: this possible mechanism may be confirmed in our sample by the significant cross-sectional association we find between reduced hemoglobin concentration and low self-reported physical activity.

The cross-sectional nature of our finding is the main limitation of this study; as the follow-up data are now being collected, a longitudinal analysis may shed light on the chronological and possibly etiological relationship between hemoglobin concentration and physical functioning. Further, although a comprehensive geriatric assessment and blood examinations were systematically performed, we did not thoroughly investigate the causes of anemia in our study population, as this would have exceeded the purpose of our study. So, it is possible that we may have missed the presence of subclinical conditions (such as cancer, gastric ulcer, bowel disease) that could have provided more insight on the relationship between hemoglobin concentration and physical function.

In sum, these findings confirm that anemia is highly prevalent in the oldest old. Further, our results confirm that low hemoglobin concentration is strongly associated with self-reported disability and reduced physical performance in the very elderly, in a way that is possibly independent of whatever is its clinical etiology. Anemia in the old population is multifactorial, and often goes undiagnosed, as approximately one third of anemia in the elderly remains of unknown origin [28]. Future research should provide more insight on the pathogenesis of anemia in this population [5, 28]. Further, as longitudinal research on nonagenarians is developing [33], the follow-up Mugello Study, as well as other studies should verify if low hemoglobin concentration in the very old will be a cause itself of adverse clinical and functional outcomes. If this will prove the case, the treatment of low hemoglobin concentration, even when the clinical etiology is not determinable or treatable in itself [34], may be considered as a mean to revert or reduce the risk of physical performance decline and of developing disability in the oldest old.
